# Origin of Pus

**Published:** 1892-01

**Authors:** J. B. Patrick

**Affiliations:** Charleston, S. C.


ARTICLE III.

### ORIGIN OF PUS.

DR. J. B. PATRICK, CHARLESTON, S. C.

Of all the topics that could possibly engage our attention, the history of pus, in the light of modern science, is the one which comes most directly to the front in the general survey of the broadest underlying progressive principle of our pathological science.

If we define the evolution of pus or suppuration to be one of the results of inflammation, to be one of its most deplorable consequences, most to be dreaded, most particularly to be prevented, then shall we look to the consummation and fulfillment of every means at command to abort the development of so sad a result.

What, indeed, is now the present order of the day in every department of the healing art? Visit the wards of the hospitals of to-day and witness the preparations made in undisguised apprehension of the advent of this formidable complication—the production of pus. See the equipments in readiness for the observance of the entire technique of antiseptic treatment. If a tumor is to be removed or an amputation to be performed; if a cataract is to be extracted, or the parturient act has just been accomplished normally and safely, behold the array of solutions, of the bichloride, carbolic acid, eucalyptus iodoform, and aristol at hand to meet the possible exigences of the occasion. With such, constituting as they do the most approved germicides at command, cavities are to be flushed, wounded surfaces irrigated, instruments and even the surgeon's hands, disinfected through repetitions of most tedious ablutions—a surgical toilette for both patient and surgeon, the purport of which would have proved wholly inexplicable to the most veteran of surgeons of a very late period.

Now, the significance of all this parade of modern methods of treatment is a resolute and determined warfare against this purulent termination of inflammation—a veritable antiseptic combat in which the enlightened surgeon contends against the outburst of *pus*, under the avowed supposition that its presence implies the presence of bacteria, while these contribute, in the opinion of many, to increase irritation of the tissues, inducing the birth and multiplication of pus cells. Whether this theory of Pyogenesis be correct, and what may be the relation that exists between the development of bacteria and that of pus, we shall not just now discuss, further than to direct attention to what, in the present state of our science, it is to be regarded as the real biological meaning of pus.

Modern research has revolutionized our views respecting the true nature of this morbid fluid. As the word pus implies, the ancients regarded it as putrefactive product from the tissues, the resultant of a general disintergation and subsequent putrescent change of the organic detritus of the part—erosive and destructive in character, breaking down health structures, resolving them into decay, and when "cradled, cribbed and confined" within restricted limitations, carrying destruction always in its wake. Another theory ascribed its evolution to a chemical change brought about by the development of sulphuretted hydrogen combining with certain ammoniacal emanations forming a hydro-sulphate of ammonia which explained the offensive odor of the product under certain circumstances. But we shall not stop to refute these antiquated views, by showing that pus is sometimes reparative, not erosive and destructive in its operations; that it is often a simple exaggerated nutritive of secretory process, contributing a healthful rather than a morbid step towards cictrization; nor shall we remind you that pus is often sweet to the taste and void of anything approximating putrescence. Neither shall I detain you with the familiar phases of its history, repeating that pus is an alkaline product of a certain specific gravity, containing fatty substances and salts; a serous fluid charged with albumen; a liquid, in a word, strikingly like, if not identical with, the serum of the blood, in which floats numbers of spheroidal corpuscles, called pus cells; that its physical appearance varies with the special variety of the product, being creamy white, sweetish in taste and smell when said to be *laudable pus*; yet often in its admixture with blood, mucus or serum, variable in its color or consistency; tenacious viscid or watery, when it is then designated as sanious, ichorous, cheesy or gummy. These facts concerning a product so studied and known require no rehearsal on this occasion. Permit me rather to call attention to the striking feature of these pus cells or corpuscles above mentioned as floating in this fluid, as they are now known to give character to the entire product spoken of as pus—a character so distinctive as to enable one to recognize pus in the smallest conceivable quantity whenever existing in the tissues or fluids of the body. No more pertinent proof could be given of this fact than to recount the remarkable diagnosis made many years ago by Dr. Donne of the incipient abscess of the breast, at a period in its development when no skill on the part of any surgeon could possibly have discovered it. Mr. Donne, while examining a specimen of human milk, at the Maternity Hospital in Paris, chanced to observe the presence of a few scattered pus cells under a microscope, mixed with the innumerable milk globules in this otherwise healthy milk, and at once declared that there must be an abscess in the breast; this woman was, however, perfectly well at the time, with no trace of trouble in the gland, yet at a short subsequent date a large abscess was actually developed, confirming Dr. Donne's diagnosis.

The cardinal point, then, in the study and recognition of the veritable nature of pus will be the interpretation of the pus cell. The corpuscular element of a fluid, when any such exists, affords the clearest evidence of the nature, origin and purpose of the product, whether this be secretory or excretory. The greatest confusion once obtained respecting the true individuality of this pus corpusule; it was affirmed that no distinction was possible between a mucus or pus globule, they were supposed to be one and the same. It was not until a forced resemblance to the white cell of the blood became generally observed that any definite idea existed respecting this question. The problem came gradually to be solved through the conjoined labors of several experimenters. Thus Addison, nearly half a century ago, pointed out the identity of pus cells and the leucocytes of the blood, and even forshadowed their migration from the blood vessels. This identity between these cells was later on again brought out by Waller in the fullest enumeration of their histological features and behavior under various reagents.

Yet these views were reluctantly accepted, since it was impossible to understand how a leucocyte, circulating with blood discs in the vessels of a part, could escape through their walls. Then came the declaration of Cohnheim, who minutely described the actual migration of leucocytes by their amæboid endowments through the attenuated walls of the capillaries within the area of the inflamed territory; and announcing that he had clearly seen the accomplishment of this phenomenon while engaged in observing the changes occurring in the web-foot of a frog, where artificial inflammation had been induced. This positive statement, however, in nowise appeared to account for the rapid multiplication of such cells in any ordinary case of profuse suppuration, but rather seemed to militate against the fact, since such a drain upon the blood as must necessarily ensue in the elimination of all of its white corpusules would at once destroy its vitality. It was then ascertained that upon the emigration of these leucocytes, these began forthwith to proliferate within the interstices of the tissues, and more than this, that they induced a similar multiplication of the connective tissue corpusules of the part; so that in due time the entire region of inflammatory action became invaded by an infinite number of rapidly multiplying neucleated cells, some of which had escaped from the vessels as emigrants within the deceased territory; while others derived from the connective tissue might be said to be natives to the manor born. This now explains fully the copious discharges of pus sometimes witnessed.

But this is not the whole of the process of true suppuration. It must be remembered that a transudation of plastic exudates from the vessels of the inflamed part was known to be one of the earliest phenomena of inflammation; now into this effusion known always to take place, leucocytes find their way. This intervacular infiltration of the plasma of the blood becomes then a fluid matrix in which nutritive material is furnished for the subsequent growth and generation of a multitudious progency of cells derived, not alone from the blood cells, but from the tissue cells also.

Pus, thus considered in its incipient or nascent development, is a product, be it observed, from the blood itself, and, indeed, resembles in its earliest stages, the vital organic processes of nutritive secretion to a marked degree. It is only when untrammelled in its process and exaggerated in its vital (though perverted) activity, that the phenomenon takes the character of a truly morbid process. Thus in following out the destiny of the separate elements of this product during their several stages, we came to a clear and perfect apprehension of the *three* distinct terminations of inflammation; by *resolution*, *organization* and *suppuration*.

First, let us suppose the initative step expressed in the effusion of exudative lymph be at once arrested, then absorption takes place before any danger reaches the tissues, and inflammation is said to terminate in *resolution*, but should the emigration of leucocytes have occured to a limited extent only conjointly with diffusible lymph and the fibrillation of its fibrin, then a slow but sure step toward tissue formation occurs, and now inflammation results in *organization*; should, however, this cell proliferation prove in the ascendency and the degenerated and metamorphosed corpusules reach their destructive influence upon surrounding parts, then does the inflammatory invasion end finally in *suppuration*.

Such is the value of modern microscopical research in unraveling the nature and origin of pus.

It seems strange that so few observers were able at first to verify this exodus of the white corpusules of the blood. Indeed Duval discussed at length the accuracy of the assertion, and ridiculed the idea of cells measuring the one-half thousandth of an inch passing through coats of the blood vessels. He believed that the preparatory step in Cohnheim's experiments upon frogs opened certain lymphatics, and the constant flow of lymph cells grouped these cells together along the outside of the vessels, stimulating the appearances which had so completely deceived him.

That no one may doubt, in the present state of our knowledge on this point, that the white blood cells do escape into the tissues through the vascular walls, we shall detail the recent experiment of Schafer as presenting irrefragable evidence of the fact.

This curious experiment consists in "feeding the white corpuscles," as Schafer terms it, and my account of the same will conclude our remarks for the present on this interesting topic.

The white corpuscles enjoy amaeboid movements, and therefore exhibit the tendencies of all amaeboid organism; that is they take in their interior any small particles of insoluble materials floating within their range.

To obtain white cells in sufficient numbers to be able to institute the curious experiment of feeding them, Schafer puts frog's blood into a small glass tube, hermetically closes both ends and lays the capillary tube aside for one hour. Examined after this period, a clot has been formed, has contracted, and the lymph around the clot is found full of leucocytes. Placing a drop of this colorless fluid from the clot, surcharged with white cells, under the microscope, he now proceeds to feed them by introducing a drop or two of Indian ink, which has been well rubbed up in a normal solution of table salt. The black particles of the Indian ink scattered through the fluid are soon engulfed into the protoplasm of the amæboid white cell, after the manner of other amaeboid organism, the intussusception occurring slowly, until almost every white cell becomes filled with little black particles which can never afterward be discharged, as these cells do not discharge their cargo.

This property of the white corpusules he thus utilizes. The insoluble particles of an ink solution he injects into the blood vessels of an animal, and then inflammation is induced at a special part. He afterwards finds in the intervascular spaces of the inflamed part, leucocytes containing similar particles of Indian Ink; the corpusules consequently must have come from the blood, for such particles have not the power of passing through the coats of the blood vessels unless carried by the white corpuscles.

In this succinct history of pus as we now understand it, I have endeavored to show and prove that, as the product of inflammation, it especially consists in the wandering from their vascular abodes of certain saline and albuminous elements; first, that pave the way for the subsequent cell extrusion, cell distention and proliferation of the white and connective tissue corpuscles and that the entire process may be regarded as an exaggeration of the normal physiological forces at play within the inflamed area of a part.—*Southern Dental Journal.*

